# Stakeholder perceptions of communication about vaccination in two regions of Cameroon: A qualitative case study

**DOI:** 10.1371/journal.pone.0183721

**Published:** 2017-08-31

**Authors:** Heather Ames, Diangha Mabel Njang, Claire Glenton, Atle Fretheim, Jessica Kaufman, Sophie Hill, Afiong Oku, Julie Cliff, Yuri Cartier, Xavier Bosch-Capblanch, Gabriel Rada, Artur Manuel Muloliwa, Angela Oyo-Ita, Awah Paschal Kum, Simon Lewin

**Affiliations:** 1 Global Health Unit, Norwegian Institute of Public Health, Oslo, Norway; 2 Department of Anthropology, University of Yaoundé 1, Yaoundé, Central Province, Cameroon; 3 Institute of Health and Society, University of Oslo, 0318 Oslo Norway; 4 Centre for Health Communication and Participation, School of Psychology and Public Health, La Trobe University, Melbourne, VIC, Australia; 5 University of Calabar, Nigeria, Calabar Municipal, Cross River State, Nigeria; 6 Faculdade de Medicina, Universidade Eduardo Mondlane, Maputo, Mozambique; 7 International Union for Health Promotion and Education, Cedex, France; 8 Swiss Tropical and Public Health Institute, Basel, Switzerland; 9 University of Basel, Basel, Switzerland; 10 Evidence-based Healthcare Programme, Pontificia Universidad Católica de Chile, Santiago, Chile; 11 Direcção Provincial de Saúde de Nampula, Departamento de Saúde, Nampula-Moçambique; 12 Health Systems Research Unit, South African Medical Research Council, Cape Town, South Africa; Rudjer Boskovic Institute, CROATIA

## Abstract

**Background:**

Understanding stakeholders’ (parents’, communities’ and health workers’) perspectives of communication about childhood vaccination, including their preferences for its format, delivery and content, is an important step towards designing better communication strategies and ensuring more informed parents. Our objectives were to explore stakeholders’ views, experiences and preferences for childhood vaccination communication in Cameroon.

**Methods:**

In 2014, in the Central and North West Regions of Cameron, we gathered qualitative data for our case study using the following methods: semi structured interviews; observations and informal conversations during routine immunization clinics and three rounds of the National Polio Immunization Campaign; document analysis of reports and mass media communications about vaccination; and a survey of parents. We conducted a thematic analysis of the qualitative data to identify themes relating to views, experiences and perceptions of vaccination information and its delivery. Survey data were analysed using simple descriptive statistics.

**Results:**

All of the parents interviewed felt that vaccinating their child was important, and trusted the information provided by health workers. However, many parents wanted more information. Parents did not always feel that they could ask questions during vaccination appointments. All participants felt that health workers and vaccination clinics were important sources of information. Social mobilisation activities such as door-to-door visits and announcements during religious services were important and accepted ways of communicating information, especially during vaccination campaigns. Information communicated through mass media and text messages was also seen as important. In general, stakeholders believed that more consistent messaging about routine vaccination through community channels would be helpful to remind parents of the importance of routine vaccination during ongoing rounds of vaccination campaigns against polio.

**Conclusions:**

This study confirms that parents regard information about childhood vaccination as important, but that health services need to be organized in ways that prioritize and facilitate communication, particularly about routine vaccination.

## Introduction

Communities’ involvement in healthcare is widely seen as essential to attaining high quality care and patient outcomes. Communication is an integral part of community participation and of healthcare delivery. Almost all healthcare involves communication with the intended recipient and often, as in the case of childhood vaccination, their caregiver [1]. An important function of communication to parents about vaccination is to provide information about the role of vaccination in their setting, vaccine effectiveness, and potential side effects. However, a major barrier to vaccination uptake for many individuals is a lack of appropriate information about these issues due to poor or inadequate communication that can negatively affect vaccination rates and undermine vaccine acceptance [2–4]. This can also lead to concerns about the trade-offs between the benefits and harms of vaccination and to fears about side effects or other consequences [5–9]. People may lack knowledge about how vaccinations ‘work’ and about the infectious diseases that vaccines prevent [5, 7, 10].

Effective communication between healthcare providers and caregivers of children has the potential to improve childhood vaccination uptake and strengthen immunization services, particularly in low and middle-income (LMIC) settings where uptake and services may be poor [11–14]. Improving communication about vaccination can be a key factor in improving vaccination outcomes [15, 16] and achieving the broader goal of knowledgeable parents and communities–important contributors to facilitating informed health choices and improving child health in many settings [1, 17, 18]. In order to plan and deliver effective communication about childhood vaccination, we need to understand stakeholders’ perceptions of communication and explore their preferences for delivering and receiving information. It is particularly important to explore these perceptions in LMIC settings [19–21] such as Cameroon, where there is little research on this topic [4].

Cameroon adheres to WHO recommendations for routine childhood immunizations [22]. According to their 2011 demographic health survey, 53% of Cameroonian children are fully vaccinated, although this ranges from 31–83% across the ten regions of the country [23]. Five percent of children receive no vaccinations at all [23, 24]. Around the time of our study, Cameroonian vaccination policy experienced two major changes. In late 2013, after several years without a case of indigenous polio, Cameroon experienced some cases of wild polio. In response to this, monthly National Immunization Campaigns against polio were organised [25, 26]. Secondly, in April 2014, the Cameroonian Expanded Programme of Immunization (EPI) introduced the rotavirus vaccine to the routine childhood vaccination programme [27]. Both of these changes influenced vaccination communication during the time of fieldwork. We observed an increase in communication activities to inform the public of the vaccination campaigns and the introduction of the rotavirus vaccine.

There have previously been reports in Cameroon of misconceptions regarding routine vaccination that were thought to have contributed to public resistance. During the tetanus toxoid (TT) vaccination campaign in the early 1990s, a rumour was spread that the vaccine would make girls infertile, which led to vaccinations being stopped [28]. This is comparable to current experiences in Northern Nigeria, where rumours link the polio vaccine to infertility [29].

In its recent 2011–2015 multi-year plan, the Cameroonian vaccination programme identified a lack of focus on routine vaccination communication. The plan cited insufficient implementation of communication interventions; low levels of ‘passion’ of health district supervisors for communication activities; low levels of financing; insufficient involvement of stakeholders such as opinion leaders, traditional leaders, and religious authorities; and a lack of training of focal communication persons [30].

### The case study objectives

The case study objectives were to explore Cameroonian stakeholders’ (health workers, parents and community members) views, experiences and preferences about:

- The communication style, medium and content of information about childhood vaccination
- The information that parents and communities want to receive about childhood vaccination

### Study setting

Our case study took place in urban and rural areas of the Central and North-West regions of Cameroon between January and May 2014. The urban setting was Yaoundé, the country’s capital, which is located in the Central region, and where 60 percent of children are completely vaccinated [23]. There, we conducted research in three health districts. The North-West region provided the rural setting for the study, with all research activities taking place in one rural health district. Eighty three percent of children in this region are fully vaccinated [23]. However, there are pockets of low vaccination completion in the hard-to-reach areas of the region.

We studied communication interventions for both routine and campaign vaccination. By routine vaccination, we mean vaccinations delivered as part of EPI following the vaccination calendar at fixed or outreach sites. By campaigns, we mean any vaccination activity that happens outside of the routine structure and seeks to reduce the transmission of particular, selected vaccine preventable diseases in an age group (of children) that is expanded for the duration of the campaign [31].

## Methods

We used a qualitative case study approach to explore stakeholders’ perceptions of communication about childhood vaccination in Cameroon. By case study, we mean a targeted, in-depth exploration of the topic with a pre-defined population within a specific geographic area [32, 33]. This allowed discussions with health workers, parents and community members about their preferences for information delivery and content and what they thought about the current strategies used in their area. Data collection settings included health facilities, district health offices, schools, churches and communities. Table 1 presents an overview of the data collection methods and participants.

**Table 1:** Overview of data collection methods and participants.

Method	Participants		Number of participants/ clinics/ campaigns*
**Interviews**	Expanded programme of immunization programme managers (n = 8)	National level managers	2
		District level managers	4
		Area level managers	2
	Health workers (n = 8)(Anyone administering a vaccine)	Fundong Health District health workers	3
		Cite Verte Health District health workers	2
		Biyem Assi Health District health workers	2
		Oyomabang Health District health worker	1
	Community members (n = 6)	Teachers	2
		Pastor	1
		Quarter Head (Local leader)	1
		Mayor	1
		Communications expert	1
	Parents (n = 56)	Rural parents	14
		Urban parents	42
**Observations**	Routine vaccination clinics (n = 5)	Rural clinics	2
		Urban clinics	3
	National polio immunization campaign (n = 3)	Rural campaigns	1
		Urban campaigns	2
**Survey of parents and caregivers**	Parents’ communication preferences during a polio vaccination campaign	Urban parents	199
**Document analysis**	Official reports and mass media communications (Television, radio, posters, banners etc.) about vaccination		

*The numbers indicate the number of participants who were involved or the number of field sites and not the total number of interviews or observation sessions.

The principle investigator (HA) and research assistant (DMN) both speak French and English, the two official languages of Cameroon. We conducted most of the interviews in the Central region of Cameroon in French, while most of the interviews in the North-West region were conducted in English or Pidgin English (a language spoken in some parts of Cameroon). DMN is from the North West Region and speaks fluent Pidgin English. She acted as a translator and led some interviews with parents.

### Sampling

We purposively selected the Central and North-West regions of Cameroon to ensure coverage of both French and English language areas, rural and urban settings, and variations in vaccination coverage.

We used convenience sampling to select parents. All parents interviewed were at health clinics with children aged 12 months or younger during a vaccination session. Sampling was influenced by how much time parents had available and their willingness to participate. The majority of parents who participated were mothers. We interviewed two fathers in one urban health district.

We used purposive sampling to select health facilities. We also used purposive sampling for participants other than parents, in order to ensure a range of participants from different levels of the health services as well as participants with different vaccination communication roles.

### Semi-structured interviews

We used interviews to explore stakeholder perceptions, preferences and experiences of childhood vaccination communication. We (HA assisted by DMN) conducted semi-structured interviews with stakeholders involved in vaccination activities, namely: health workers, parents and community members. We conducted the interviews in vaccination clinics, offices, churches and schools. (See Appendix 1 for interview guides)

### Participant observation and informal conversations

We carried out observations and informal conversations during routine immunization activities at health clinics and during three rounds of the National Polio Immunization Campaign in community settings. These observations complemented what was said in interviews with data on what was taking place ‘on the ground’. During these observations, we also conducted informal conversations with health workers, social mobilisers (lay health workers trained to deliver health promotion messages) and parents. These conversations allowed us to talk in a more relaxed manner about participants’ views of the vaccination communication strategies they were delivering. The focus of our observations was on communication in the vaccination setting, the interactions between the various groups involved and the content of the information given about vaccination. At the beginning of fieldwork, we used a structured observation guide based on the EPI guidelines for Cameroon to record vaccination interactions in the clinic. After we became comfortable in the setting and understood how the vaccination sessions worked, we moved to taking free observation notes. During vaccination campaigns, we kept field observation notes in a field journal.

### Survey of parents and caregivers

We carried out a survey with 199 caregivers in the Oyomabong area of Yaoundé during the April round of the polio vaccination campaign. The majority of the caregivers surveyed were mothers. We also spoke with fathers, siblings and other relatives. We conducted the survey opportunistically to make use of the interaction with caregivers as part of our observation of a campaign. There was no randomisation or sample size calculation since our aim was not to obtain representative estimates with a given precision. During the course of the two days, we administered the survey to as many as possible of the households to which a vaccination team delivered a polio vaccine. Our survey included questions addressing how they had heard about the vaccination campaign for polio and the introduction of the new rotavirus vaccine and what their preferred communication channel would be. We partially based the survey questions on a discussion with the EPI office about the kinds of information that would be useful to them. After the vaccination team had spoken with the household, we carried out the survey verbally with the caregiver and recorded the answers on a standard form. (See Appendix 2 for the survey questions)

### Document and media sources

During fieldwork, we collected media articles and stories about vaccination, and vaccination-related items such as child health cards, posters or banners, that related to the study objectives. We read newspapers, watched the daily news and visited a rural radio station to receive copies of their programming about vaccination. This allowed us to compare coverage in the popular media with what stakeholders were saying about vaccination information available in the public sphere.

### Data analysis

We transcribed interviews during fieldwork and after leaving the field. After transcription, we used a thematic analysis approach [34]. We coded each interview transcript and grouped codes into categories based on commonalities and patterns in the data. Next, we grouped the categories together based on similarities to form themes. Finally, we coded the data from the observations, document and media sources using these categories and themes. In our findings section below we present themes and categories according to findings about stakeholders’ views and experiences of childhood vaccination communication, findings specific to communication and information in healthcare settings and findings specific to communication and information in community settings.

We used simple descriptive statistics to analyse the survey data.

### Ethics

La Comité National d’Ethique de la Recherche pour la Santé Humaine (CNERSH) granted ethical clearance for this study in Cameroon. We submitted the study for approval to the Norwegian Regional Committee for Medical Research Ethics. They concluded that the project fell outside of their remit.

All interview participants signed an informed consent form or agreed orally to participate after we had explained the consent form. The people in charge of health facilities and campaigns granted access to observe vaccination sessions and campaigns.

## Findings

We present our findings in three parts: general findings about stakeholders’ views and experiences of childhood vaccination communication, findings specific to communication and information in healthcare settings and findings specific to communication and information in community settings.

### Findings about stakeholders’ views and experiences of childhood vaccination communication

#### Parents perceived vaccinations as important, but questioned repeat vaccinations

All of the parents we interviewed had followed the vaccination schedule and believed that vaccination was important for the health of their child. The majority of parents interviewed did not see taking their child for routine vaccination as a big decision, but part of everyday life.

This perception of vaccination as normal was demonstrated by parents who could not conceive of a parent not vaccinating their child. Most did not know anyone who had not vaccinated or had dropped out of the vaccination programme. However, after multiple rounds of the polio vaccination campaigns parents started to question the need for their child to be vaccinated repeatedly. Parents knew that vaccinating their child was important but were confused or worried about why they were vaccinating or had to vaccinate so many times.

#### Receiving information about vaccination

**I think I have enough but I don’t know what I am missing.** Many parents reported that they had not received any information about vaccination, whereas other parents felt they had enough information about vaccination and did not want any more. Interestingly, when we probed later in the interviews to find out what information these parents would like to have about vaccination, many came up with a number of questions.

*I don’t feel like I know all about vaccination but I am aware it is important*. *I want to know what will happen if you fail to vaccinate your child*. (Urban Parent)

Some parents explained that they felt they had enough information because they did not know what information they were missing. For example, all stated that vaccinating their child was important but many did not know why:

*The truth is that I have been hearing about vaccinations but I don’t really know whether… it is only when I was pregnant that I knew why I was taking medicine*. *But now I don’t even know why I am vaccinating my child*. *It is only polio that I know that they vaccinate the child so that some parts of his body won’t be weak when they are growing up*. *Apart from that*, *I don’t know about any other vaccine*. *I don’t really know why they vaccinate*. (Rural Parent)

**I want more information presented in a clear and simple way that I understand.** Parents liked receiving information about vaccination and wanted more information than they were receiving, presented in a clear and simple way, in a language that they could understand and reflective of their local cultural and linguistic context. An example of this was the posters distributed for the vaccination campaigns. They were sometimes received in the wrong language or with the wrong dates. Parents wanted to know more about the side effects of the vaccines and how to treat them; when to come back for their next appointment; and the importance of following the vaccination calendar. They felt that the information that they were being given was too general and did not provide them with the details they wanted to know.

#### Health worker perspectives on vaccination information

**Information health workers thought parents should know.** Health workers felt that it was important for parents to know when to come back for the next appointment and to understand the importance of vaccination:

*I think the most important information is to let the mothers know the importance of vaccination yes… I think that a mother has her child and they should know why it is important to vaccinate that child*. *You will vaccinate that child*. (Rural health worker)

Health workers felt that if a parent returned on time for their follow-up appointment it showed they were well informed:

*When you see a mother who comes back*, *it means that she is well informed about vaccination and she knows the importance of vaccination*. (Urban health worker)

**What is my job as a health worker when it comes to communicating about vaccination.** Health workers believed that their job was to communicate the importance of vaccination in order to convince parents to come for the vaccines. In most cases, parents accepted this as they felt the health workers had their best interests at heart. However, we observed some cases, especially during campaigns, where parents felt pressured to vaccinate their children. We observed that they were given little time to make the decision. In some cases, children who vaccination teams met on their way to school were vaccinated without parents present. Older siblings often gave consent for their younger siblings in this case.

#### Sources of vaccination information

Parents mentioned a number of sources from which they had heard about vaccination. However, they said their preferred sources from which to get vaccination information were the clinic, door-to-door visits from social mobilisers, TV, and radio (See Table 2).

**Table 2:** Preferred source of information as mentioned by parents during interviews and the survey.

**Health services sources**	
Clinic, hospital or nurse	45
Health talk	1
Vaccination book	1
**Social mobilisation sources**	
Door to door	24
Church	6
Social mobilizer	2
Campaign	2
Quarter Head	1
**Mass media sources**	
Television	104
Radio	35
Media	21
Text message/telephone	17
Newspaper	5
Poster	4
Books	2
Internet	1
**Personal sources**	
Friend or family	2

### Communication and information in healthcare settings

Parents said they had received information about their children’s vaccinations in healthcare settings at various time points including antenatal care, delivery, or at the first vaccination appointment. Routine vaccination information was mostly only available in healthcare settings. Health talks given during vaccination appointments were the main source of information for most parents followed by child health cards, SMS reminders and conversations with health workers. In this section, we will present our findings related to the main clinic-based communication interventions; child health cards, group health talks, text message and phone reminders, and health workers.

#### Child health cards

**An important tool and reminder.** Parents felt that the child health card was an important vaccination information tool as it included information about when children should be vaccinated and allowed health workers to record the time and date of their next appointment. It served as an important reminder for parents. Most parents had to pay for the card before their child received their BCG vaccine at 1–7 days old. In Cameroon, at the time of fieldwork, there were two different child health cards in use. One was the traditional child health card that recorded vaccinations and weight on a single paper. The other was a more in-depth child and maternal health book that included a section on vaccination. This book was being piloted in two health districts (Biyem Assi (French-speaking) and Santa (English-speaking)). Parents liked the new book but were angry that it was expensive (up to ten times the cost of the normal card). Most said that the information on the child health card, whether it was the old card or the new book, had only been explained to them once during their first vaccination appointment

**A source of frustration.** The child health cards were a source of frustration for some parents. If a parent forgot their card, they could be sent away and told to come back with their card during the next vaccination session. Parents were frustrated when health workers forgot to write the next appointment in their cards, or when the date written for their next appointment was a holiday or a day when the clinic was closed. This meant extra trips to the vaccination clinic and a potential loss of working hours.

**A way of organizing appointments for health workers.** We observed that health workers used the cards to triage and organize parents upon arrival at a clinic for routine vaccinations. It was often unclear at the clinics where parents were supposed to put their cards. If it was put in the wrong place this could lead to confusion and sharp verbal corrections from health workers or other parents, sometimes leading to feelings of embarrassment or intimidation. The health workers used the cards to call parents for vaccination in the order they arrived. However, this was not always successful and parents got frustrated if called in the wrong order. The health workers also used the cards to determine if there were enough children to open a vial of vaccines. If the number of cards was inadequate to open a vial, they sent parents away and told them to come back on the next clinic day.

#### Health talks

We observed that most of the information that parents received at the clinic came during the group health talk. Parents and health workers confirmed this observation during interviews. We observed that health talks at the clinic were given to parents in plenum in the waiting area; or were given to small groups of parents who were called into a separate room. When health workers gave health talks in the waiting area, parents who arrived late missed the information and consequentially received no information at the appointment.

**Long waits for health talks to begin.** Our observations and interviews found that parents frequently had to wait for a long time for the health talk to begin, sometimes up to two hours. We observed that the length of the health talks varied from clinic to clinic, the shortest was two minutes and the longest over an hour. We observed that parents became visibly distracted and lost concentration during longer talks. Parents also mentioned that they could not concentrate on listening to information while trying to entertain their child and keep them quiet. A second distraction was the practice of giving the oral polio vaccine during the talk. Parents found it difficult to continue listening while their child received the vaccine or while the health worker moved around the room. In some instances, we observed that the content of the health talk did not cover vaccination at all.

**Choosing what to talk about; the health workers choice.** The health worker administering the vaccinations was often the one giving the talk. A health worker explained that she could decide on the topic for the day and the length of the talk:

*You choose your topic before the day of the vaccination*. *You make a brief something on what you are going to say to the mothers*. *Then you come and you lecture*, *like I did*. *You make the women sing*. *It is always important to make the women sing a song so that they can become lively and active*. *Then you go ahead with your talk*, *you give them a chance to ask questions*, *if they have any doubts*. *They ask and then you answer*. (Rural health worker)

From our observations and our interviews, we found that the content or timing of these talks did not follow a standard procedure. Health workers said that they believed that health talks were the easiest way to inform parents but felt they could be more effective educators if they had access to teaching aids. They wanted information flip charts or storyboards to show the implications of vaccine-preventable diseases. They also felt that pamphlets with information from the health talk would be useful to send home. The posters that were hanging in the clinics were often old and out of date.

**Shying away from asking questions.** Many parents we spoke to felt uncomfortable asking questions or did not know they could ask questions during appointments or the health talk. Health workers also recognised that parents did not ask many questions.

Some parents were too shy to ask in front of a group during a health talk or said that another parent had asked the question they were thinking of. In rare cases, parents would ask a question and be ignored by the health worker. Other parents said that they felt that health workers were completely open to questions and would not have a problem asking.

#### Respect our time and don’t make use wait

Many felt the clinics could be more time-conscious, with some parents waiting for up to four hours to receive their vaccinations. Nor did parents like it when other parents came late as they felt it held up everyone, given that vaccinations often did not begin until everyone had arrived:

*The clinic is good but they don’t respect our time (*Urban parent)

*A parent might come here very early in the morning just to weigh her child then you sit until twelve o’clock*. *As for me*, *I have already weighed my baby and now I am waiting to vaccinate because I want to go home as soon as possible*. *So they are not fast at all*. (Rural parent)

Finally, after delivering the vaccines, health workers returned the child health cards and told the parents when to return for their next appointment. At this point, we observed parents trying to balance a half-dressed, crying child, the child’s clothes and their own personal belongings. Their focus and attention were on comforting their child and trying to leave the clinic, not on listening to the information from the health worker.

#### Vaccination reminders via text message and telephone

Parents wanted phone reminders from clinics about their vaccination appointments. Some had seen this happen in a Nigerian movie. However, urban parents felt that the main barrier to text message reminders was cost, as the health worker would have to pay for the text message out of their own phone credit, as there was no government support for such a service.

*Text message here would not work*. *No*, *people are not willing to sacrifice their credit*. *It would work in private clinics but not government*. (Urban parent)

*If we call a mother who left and did not come back*, *we call with our own money and it is us who lose*. *You have to take some money out of your taxi money to call a mother*. (Urban health worker)

Health workers also suggested text messages as a way of improving attendance at vaccination sessions and make it easier to follow up with children who had missed an appointment.

#### Health workers as a source of information

**I trust my health worker.** Both parents and health workers said that health workers are an important and trusted source of information for parents. The majority of parents trusted the information they received at the clinic because of the education and experience of the health workers. When we asked parents whom they would turn to with questions about vaccination, they invariably answered their healthcare provider.

Parents felt that they could hold health workers accountable by returning to them at the clinic if something went wrong with the vaccination. Two parents told stories of rejecting advice from family and friends about treating vaccination side effects, as it contradicted the information they had received from the clinic.

*(After) the last clinic I attended*, *I couldn’t sleep at night because the child was crying*. *Some people proposed that I should apply kerosene and I said no*, *that the child will cry and cry and stop*. *Some said I should put the child in cold water and I still rejected*. *Some said I should put honey and I said no*, *I wasn’t told at the clinic to do so*. (Rural parent)

**Health workers believe they are an important source of information.** Health workers believed that the clinic was an important source of information for parents and was the best location to communicate to parents about vaccination. However, they felt that this was not the case with fathers as they rarely attended vaccination appointments. Health workers felt that fathers generally heard about vaccination at church, in their community or from their wives. Informing mothers could therefore help to inform fathers.

**Parental preferences for their interactions with health workers and the clinic setting.** Parents wanted health workers to be patient and not to hurry. They felt that a good health worker told them who they could talk with about their problems and was quick to react and to provide appropriate follow-up care. They liked clinics that were clean, had enough room for everyone to sit and wait, had energetic, punctual, informative and caring staff, was well organized, and did not have vaccine shortages. If they found a clinic they liked they returned there to complete their child’s vaccines.

*I really like it*. *I like that it is clean and they are welcoming*. *I never have any worries*. (Urban parent)

*At this hospital*, *they are not really informed*. *They didn’t really give any detailed information here about vaccines*. *In the general hospital*, *they tell you the vaccine and inform you whether to take it or not*. *But the pitfall is that they don’t have vaccination at all times*. (Urban parent)

Parents interviewed felt that not all of the health workers were good. They wanted them to be more caring and not to yell at parents in an “uncaring way”. Parents said that they did not mind being scolded or yelled at by a nurse if he/she felt they had done something wrong as long as the nurse was caring and took the time to explain what the issues were.

### Communication and information in community settings

Communication and information about vaccination available in community settings were mostly related to vaccination campaigns. Information was disseminated through mass media and social mobilisation activities. Stakeholders, both those receiving and providing the information, wanted information to be available through a wider range of community settings. They also felt that more information about routine vaccination could be disseminated through the channels used for vaccination campaign information.

#### Mass media and government sources

**We trust the government.** Parents in Cameroon generally trusted government sources of information. They saw them as credible and believed that the government would not hurt their own people. Health workers also trusted the government’s motives. One strategy used by the government was the mass media, used mostly for vaccination campaigns. Parents trusted the programmes produced by the Ministry of Health on radio and television. They liked seeing what the disease looked like and that the programmes raised awareness about vaccination campaigns:

*A programme under the Ministry of Health*, *yeah then I would trust the information*. *For example*, *this vaccination for polio*, *they were coming house by house*. *If I didn’t have it from the TV*, *I would not allow them to touch my child because I knew in listening to the TV that there would be a programme of vaccination*, *they would be coming to my door to vaccinate children*. (Rural parent)

**Timing of vaccination messages hinders reaching the target audience.** Parents felt that the communication sources used were appropriate but that the timing of messages could be improved. For example, mothers felt that messages on TV were played at the wrong times, for example during the news when they were preparing dinner instead of during soap operas in the afternoon. All stakeholders felt that TV and radio should be used more frequently for information about routine vaccination and that there was a lack of follow through and repetition in the mass media about vaccination messages. This lack of repetition of messaging about childhood vaccination meant that they were not as successful as they could be. Radio was preferred in rural areas where many households did not have televisions.

**Vaccination messages need to be repeated.** Health workers, parents and community members felt that messaging from the Ministry of Health and local health centres could be more consistent in relation to both timing and content for routine vaccination. They highlighted that new parents enter the vaccination programme throughout the year and need to receive information about childhood vaccination for the first time. They felt that the sources used for vaccination campaigns could be employed to continue carrying the message about the need for routine vaccination after campaigns were over. For example, they felt that improved communication was achievable by focusing more consistently on churches, not just during campaigns. Health workers believed that communication needed to be constant and reinforced in order for the message to be remembered.

*To improve communication about vaccination I think it is just constant sensitization*, *constant*, *constant sensitization… because you see the human being is some sort of person who forgets at times although he knows*, *when you constantly tell him*, *tell him*, *tell him he will retain but if you just tell him once and go he will also be slack about it*. (Rural health worker)

**Text message from the Ministry of Health for the launch of new vaccines. The problem of literacy and cell phone ownership.** During the observation period, the Cameroonian Ministry of Health sent a text message to all public users of certain cell networks to inform about the launch of the rotavirus vaccine. Parents felt that receiving a text message was an easy way to receive information but that it was not widely used. However, rural parents raised challenges around informing and reminding about vaccination via text message as they thought that lower literacy levels and rates of cell phone ownership would limit the number of parents reached:

Interviewer: So for you what could hinder using text message?

*Mother*: *People who don’t know how to read will be the problem*. *That some people have phones but they can’t read like the grandmothers and the grandfathers and that the way they put their names in the phone they arrange it in such a way that they know which comes after which*. *And if you are not able to read the information you wouldn’t be able to understand unless you have someone who can read it for you*. *At times the messages won’t even go to everyone (network problems etc)* (Rural Parent)

#### Social mobilisation activities

Social mobilisers (community members trained to deliver health promotion) were an important source of information for parents, especially about vaccination campaigns. Social mobilisers’ training in Cameroon instructs them to be open to questions and ask if parents have questions during social mobilisation activities such as door-to-door visits, activities in town squares, talking with community leaders, making announcements over loudspeakers and training women’s community based organisations about vaccination and how to talk with other women.

**Social mobilisers; important partners in spreading the vaccination message.** Health workers, especially those in rural areas, believed that social mobilisers were an important support for the mass media messaging.

*Yeah*, *like there is normally the TV and the radio but all that the information does not go to all the places*. *There are places without TV and there are places without radio even as we are in this environment there are deeper villages who will not get the information over the radio and the tv so we work in collaboration with community workers*. *We have community workers from all the communities that we train here and we send them out to do some sort of social mobilisation*, *yes*, *to mobilise the population*. *They tell them what they have been taught here that they should vaccinate*, *reasons why they should vaccinate*, *and all that*. (Rural health worker)

In rural areas, the social mobilisers were more visible and known within the community and were often the only source of information about the vaccination campaigns.

**The importance of including religious communities.** Another important avenue for social mobilisation was churches, particularly for informing fathers. People trusted their churches and announcements made there. Health workers also believed that church announcements were important for informing people about vaccination and some went as far as making announcements in their own churches.

Social mobilisers worked with religious leaders to make announcements during religious gatherings. The religious leaders agreed to allow vaccination teams to come to the churches during vaccination campaigns. Some religious leaders received training in how to talk to parishioners about vaccination. In rural areas, participants considered the church as the key information source given that the vast majority of the population regularly attends religious ceremonies.

*For me I don’t listen to the radio*. *I am not used to the radio but it is important to announce in churches on Sundays (*Rural parent)

### Survey findings

Only 32% of parents interviewed had heard about the new cases of polio in their area. Sixty-eight percent of parents did not know that the vaccination campaign would be coming to their door that weekend. Forty-seven percent of parents surveyed did not know which disease the vaccine their child was receiving was for.

One hundred and eighteen parents (59%) surveyed had heard of the new vaccine that was being introduced into the Cameroonian EPI programme. Of these 118, sixty-three (53%) new that the vaccine was for rotavirus or some sort of diarrhoea.

For both the vaccination campaign and the new vaccine, the majority of parents had received information through mass media sources such as television and radio.

## Discussion

Our findings illustrate that health workers, parents and community members are interested in communication about childhood vaccination and were able to share a wide array of experiences and perspectives. In our findings, we provide examples of when participants felt that communication about vaccination worked well and cases where it could be improved. There were no major distinctions between urban and rural participants in their preferences for amount of information. There were differences in the ways in which they would like to receive information about vaccination. Rural parents preferred radio and announcements during religious meetings whereas urban parents had a preference for television and text messages. All parents liked receiving information from health clinics and social mobilisers.

While health workers tended to speak to parents in general terms about the importance of vaccination, parents wanted a more in-depth understanding of why they were vaccinating, what the risks and benefits were and how the vaccine would affect their child. A recent qualitative evidence synthesis, along with studies conducted in Nigeria and Bangladesh, had similar findings [4, 19, 20]. These studies also found that health workers were the most important source of information for parents and parents had specific expectations of their interactions with them [4, 19, 20]. Parents in all these settings wanted health workers to be caring and compassionate and to take their time [4]. When these expectations are not met, parents’ perceptions of vaccine services, their interactions with health workers and in some cases their intention to vaccinate may be affected [4].

Participants expressed views about specific channels of vaccination communication. For instance, they suggested that mobile health (mHealth) communication strategies, such as appointment reminders via text message, are acceptable and potentially preferred methods of communication. Related COMMVAC studies in Nigeria and Mozambique found a preference for text message reminders, especially amongst urban populations [20, 21]. Other studies have found that text messages are acceptable amongst parents for receiving health information and reminders [35–38]. Text message reminders could be a viable option for sending out information about vaccination but would most likely need to be supported by other interventions that provide opportunities for vaccine-hesitant parents to discuss their concerns[4]. Advances in social networking and online platforms, as well as in network access, mean that mHealth strategies can now be used to promote discussion about vaccination, and not just to deliver static, unidirectional messages (Obregón and Waisbord 2010). Although this would be difficult in rural Cameroon currently, these approaches are now in use in many LMIC settings–for instance, Medinfi in India gives reviews and recommendations for doctors (http://www.medinfi.com/). Some key m Health challenges remained unsolved, including investment, running and maintenance costs; equity issues, in terms of participation among poorer groups and coverage of hard-to-reach areas; and ensuring that parents have the right to object.

One of the issues raised by health workers, parents and community members in this study was the need for constant and consistent messaging concerning the importance of routine vaccination. Stakeholders believed that messaging should be consistent over multiple sources (TV, radio, community announcements) and that there should be a focus on continuing such messaging after vaccination campaigns, to ensure that routine vaccination was not neglected.

This study identified social mobilisation strategies intended to inform and educate parents and communities as important to stakeholders, especially in rural areas. Two of the key interventions mentioned were announcements at religious ceremonies and door-to-door visits during vaccination campaigns. In their study of social mobilisation during polio eradication campaigns, Obregon and Waisbord highlight the need for social mobilisation strategies to take a bottom-up approach that engages the local community and leaders and is tailored to individual settings. Social actors, they argue, should be seen as more than people repeating a message but as engaged members of a changing community that can play a larger role in communication efforts [39]. A SR of community-aimed interventions for vaccination communication [13] found very few studies, and the effect of some kinds of social mobilisation are therefore still uncertain. However, the review suggests that community-based structured discussions of the pros and cons of vaccination, with the purpose of informing decision making, can have a positive effect on vaccination uptake [13, 40].

### Strengths and limitations of the study

The main strength of the case study was the iterative and flexible process that we adopted when conducting fieldwork. An example of this was the decision to add a survey with parents during a vaccination campaign to see if our findings from interviews in clinics were similar when we interviewed parents in the community. Another strength was the collection of data among different parts of the population, including people from different regions of Cameroon, from urban and rural settings, with different levels of vaccination coverage, and from different cultural/ethnic/language groups. A potential limitation of the study is that it was conducted during a polio epidemic where there was an increased focus on campaign activities. This could have influenced the data we collected, particularly the extent to which campaigns were the focus of vaccination communication activities and received priority over routine vaccination activities. Another limitation was that we did not interview caregivers who had decided not to vaccinate or had dropped out of the vaccination programme. We do not know how their views differ from other parents.

## Conclusions

This study has explored parents’, community members’ and health workers’ perspectives of how information about childhood vaccination is communicated in two regions of Cameroon. We found that communication about childhood vaccination is important to health workers, parents and community members. However, at the moment, health systems are not catering to stakeholder preferences in relation to content and source. We observed that vaccination clinics were not organized in a way that prioritises or facilitates communicating with parents. In general, stakeholders believed that more consistent messaging about routine vaccination through community channels, such as religious services, would be helpful to remind parents of the importance of routine vaccination during ongoing rounds of vaccination campaigns against polio. It is important that health systems focus on meeting stakeholders’ communication needs by focusing on what is being done well during vaccination campaigns and carrying this over to communication about routine childhood vaccinations.

This study contributes to the currently very limited body of evidence from LMICs on stakeholders’ perspective on how information on childhood vaccination is communicated [4]. The findings may help policy makers and programme managers in the field of vaccination to better understand the information needs of parents and caregivers, and how these needs can be addressed.

## Appendix 1: Interview guides

### Interview guide: Parents

What brought you to the clinic today? / How did you hear about the campaign?

Ask about the decision to immunize or not

- Who influenced or took part in the decision to immunize the baby
- Was the decision made the same way for all of your children
- What kinds of information and support did you receive to make the decision?
- Do you feel like you were missing any information to make the decision?
  ○ If missing then where would they have liked to get this information from
  ○ What exactly did they feel they were missing i.e. info about side effects, calendar etc
- What kinds of information have you received since you made the decision to immunize?
  ○ What were the sources of the information and / or support? (types of media, friends, family etc)
- What did you think of the information and / or support that you received?
- What kinds of information and / or support would you like to receive?
  ○ In what ways would you like to receive this information or support (how would you like to be communicated with)?

Clinic experiences

- Can you tell me about your experiences with clinic where you have vaccinated your child?
  - Attitudes and behaviour of health care providers
  - Information received and manner in which it was presented
  - Physical environment
  - Access
  - Waiting times
  - Quality of the care received

Questions about the polio campaign

- How do you feel about how information was delivered during the recent polio vaccination campaign?
- Is this different from routine immunization?
- How do you prefer to receive information about vaccination?

Questions if not covered in answers above

- What do you think is the point / purpose of vaccination?
- Did you receive any information about side effects of vaccination?
- Did you receive any information on when children should not be vaccinated (contra-indications)
- Were you told when to come back? Were you given a specific date?

Partially vaccinated

- What are the reasons for your child not being vaccinated according to schedule? (probe for some of the common issues, if needed, including lack of information)
- What are your plans in terms of immunizing your next child? Explore the reasons

### Interview guide: Community members and other stakeholders (community and religious leaders, chairs of local health committees, women’s group leaders, traditional healers)

- What is your role in health activities in this area / village?
- Please describe your role within vaccination in your community
- Can you please tell me about how you became involved in vaccination work in your community
- Who do you communicate with about vaccination in your setting / community?
- Can you please describe how you communicate with these people about vaccination
- Do you also assist with vaccination campaigns?
  ○ If yes Is your role different during campaigns (where relevant)?
- Can you please describe any other information and support that is used to improve childhood vaccination uptake in your settings (e.g. through schools)?
- *For each communication intervention*, *please describe*:
  ○ The content of the communication interventions
  ○ The frequency with which it is delivered and the format/s used
  ○ Who delivers the intervention
  ○ Who the communication intervention is targeted to
  ○ Whether the intervention is used in combination with other interventions
  ○ With which vaccines the intervention is used
- For the main vaccination communication interventions that are used in your setting, what has worked well?
- Where have you encountered problems in implementing these interventions?
- In your view, what might help delivery vaccination or improve uptake here? What might help improve communication with caregivers about vaccination?
- What other issues may be important in spreading vaccination information in your setting?

### Interview guide: Programme managers

Background and Demographic information

- ‘Official’ title
- Place of work and duration of work in that position
- Describe how you came to be involved in the vaccination programme?
- Please describe what you see as your current role within the vaccination programme
- How long have you worked in the area of vaccination delivery?
- Are you involved with any vaccination committees?

Communication interventions

- How does the health system tell / inform parents and the public about vaccination in your setting?
- Do you have a name for these types of activities in your setting (e.g. social mobilization activities, IEC activities)? In our project, we call them………
- In your system, who is responsible for:
  ○ Developing communication activities
  ○ Delivering communication activities
  ○ Managing communication activities
- Please describe any communication interventions that are being used to improve childhood vaccination uptake in your settings
- For each intervention, please describe:
  ○ Are these applicable mostly for routine immunisation or Mass campaigns or both?
  ○ What are the current programmes in which communication strategies are used commonly?
  ○ The content of the communication interventions
  ○ The frequency with which it is delivered and the format/s used
  ○ Who delivers the intervention
  ○ Who the communication intervention is targeted to
  ○ Whether the intervention is used in combination with other interventions
  ○ With which vaccines the intervention is used

**Start by noting the respondents’ responses and once s/he has finished, *use the COMMVAC taxonomy to prompt for any further interventions(to start filling in the in country taxonomy)*. *Also note that need to tailor this question to the role of the respondent within the health system*

- Probe the extent to which these interventions are being implemented at scale / in practice, by asking where they think they are doing well and where there are problems in relation to communication for vaccination
- Find an example of a communication intervention for which scale up has been attempted, and probe what issues arose in trying to scale up
- Do you have any suggestions on how to improve information delivery regarding vaccination in the country? (i.e. to meet the goals set for communication in your setting) Are there any laid down policy documents on how vaccination communication strategies should be carried out (at each level of government)?

### Interview guide: Vaccinators (includes lay health workers, nurses, other mid-level providers, mobile brigades etc.)

Demographic and other descriptive information

- Health cadre title
- Place of work and duration of work in that location

Background information

- How did you start working in vaccination? / Could you tell me about your work here at the clinic?
- How long have you been working:
  ○ in vaccination delivery?
  ○ at this vaccination clinic?
- Please describe your role within the vaccination programme / as a vaccinator

Vaccination training

- Can you tell me about the training you received to work on vaccination?
  ○ What this included?
  ○ Whether the training/s included anything on communication
  ○ When the training was received
- What sort of materials, such as manuals, do you have to support your work?
- During supervision visits, do you receive any support around communication with caregivers?

Introduction to the vaccination activities

- Please describe what you usually do when running a vaccination session / what happens in the clinic on an average clinic vaccination day
  ○ How the vaccination is organised
  ○ How many caregivers are usually seen

Communication interventions 1

- What sorts of information do you / your colleagues / the clinic share with caregivers regarding vaccination? [*Note that we want to find out about the content and format of the different interventions*]
- For each intervention, please describe:
  ○ The content of the communication interventions
  ○ The frequency with which it is delivered and the format/s used
  ○ Who delivers the intervention
  ○ Who the communication intervention is targeted to
  ○ Whether the intervention is used in combination with other interventions
  ○ With which vaccines the intervention is used

**Start by noting the respondents’ responses and once s/he has finished, use the COMMVAC taxonomy to prompt for any further interventions**

Communication interventions 2

- For the main vaccination communication interventions that are used in your setting, what has worked well?
- What challenges / problems do you encounter during this delivery of vaccine information? What discourages you? [*Could include job satisfaction*]
- What are the things which encourage you during this delivery?
- What other issues may be important in implementing vaccination communication interventions?
- Resources available to support these activities

Views regarding information and communication

- How do you feel about giving information during vaccination?
- Which information do you think is the most important for parents to know?
- What do you think is the easiest source of information for parents?

Relations with community groups

- Are there structures / committees in the community around the clinic to which you relate / in which you participate?
- How do you liaise with important groups in the community?
- Are their important people/ groups I should speak to in the community regarding vaccination and child health?
  ○ Probe for people / groups that are both in favour of and against vaccination

## Appendix 2: Survey questions (translated to English from French)

- Do you know which disease the vaccine we are giving today is for?
- Have you heard about the new cases of polio in the central region?
- Do you know that there was going to be a vaccination campaign this weekend?
  ○ If yes, how did you find out?
- How would you like to hear that there is going to be a vaccination campaign?
- Have you heard about the new vaccine for infants aged 0–11 months?
- Can you tell me what disease this new vaccine is for?
- How did you hear about the new vaccine?

## Supporting information

S1 File Survey results for communication about the polio vaccine. (PDF)

S2 File Survey results for communication about the rotavirus vaccine. (PDF)

S3 File Survey data. (XLSX)

S4 File Observations of the polio campaigns in public places. (PDF)

## Abbreviations

COMMVAC: The Communicate to Vaccinate Project
EPI: Extended programme on immunisation
UNICEF: United Nations Children’s Fund
WHO: World Health Organization
