# Supplementary material for: Stakeholder perceptions of communication about vaccination in two regions of Cameroon: A qualitative case study
Source: PLoS One. 2017 Aug 31;12(8):e0183721. doi: 10.1371/journal.pone.0183721 (PMC5578665; doi:10.1371/journal.pone.0183721)
Supplement: S1 File — (PDF) [file pone.0183721.s001.pdf]

Mini survey results polio

Day 1

Total asked: 116

1) Do you know which disease we are vaccinating against today?

Yes	68	59%
no	48	41%

2) Have you heard about the new cases of polio?

Yes	33	28%
no	83	72%

3) Do you know that there was going to be a campaign this weekend?

Yes	30	26%
no	86	74%

4) If yes how did you hear about the campaign (some mentioned more than one source)

Clinic	Don't know	Media	Didn't answer	Neighbourds	Radio	School	Tv	Door 2 door
3	1	2	2	1	7	1	12	1

5) What is the easiest way to inform you about the vaccination campaigns? (some mentioned more than one source)

any	campaign	Church	Clinic	Don't know	Door 2 door	Media	Newspaper	Poster	Radio	Sms	Telephone	TV
1	1	3	11	7	13	13	2	3	19	4	5	55

Day 2

Total asked: 83

1) Do you know which disease we are vaccinating against today?

Yes	37	46%
no	46	54%

2) Have you heard about the new cases of polio?

Yes	31	37%
no	52	63%

3) Do you know that there was going to be a campaign this weekend?

Yes	33	40%
no	50	60%

4) If yes how did you hear about the campaign (some mentioned more than one source)

Clinic	Announcement	Market	Don't know	Media	Didn't answer	Neighbourds	Radio	School	Tv	Door 2 door
1	1	1	0	5	0	2	8	0	14	2

5) What is the easiest way to inform you about the vaccination campaigns? (some mentioned more than one source)

any	campaign	Church	Clinic	Don't know	Door 2 door	Media	Newspaper	Poster	Quarter head	Radio	Sms	Telephone	TV	Word of mouth
2	0	1	1	4	11	8	0	0	1	13	5	3	39	2

Total Day 1 and 2

Total asked: 199

1) Do you know which disease we are vaccinating against today?

Yes	105	53%
no	94	47%

2) Have you heard about the new cases of polio?

Yes	64	32%
no	135	68%

3) Do you know that there was going to be a campaign this weekend?

Yes	63	32%
no	136	68%

4) If yes how did you hear about the campaign (some mentioned more than one source)

Clinic	Market	Don't know	Media	Didn't answer	Neighbourds	Radio	School	tv	Door 2 door
4	1	1	8	2	3	15	1	26	3

5) What is the easiest way to inform you about the vaccination campaigns? (some mentioned more than one source)

any	campaign	Church	Clinic	Don't know	Door 2 door	Media	Newspaper	Poster	Quarter head	Radio	Sms/ Telephone	Tv	Word of mouth
3	1	4	12	11	24	21	2	3	1	29	17	94	2
