# Supplementary material for: Stakeholder perceptions of communication about vaccination in two regions of Cameroon: A qualitative case study
Source: PLoS One. 2017 Aug 31;12(8):e0183721. doi: 10.1371/journal.pone.0183721 (PMC5578665; doi:10.1371/journal.pone.0183721)
Supplement: S2 File — (PDF) [file pone.0183721.s002.pdf]

Mini survey results Rotavirus

Day 1

Total asked: 116

1) Have you hear that there is a new vaccine for babies 0-11 months?

Yes	65	56%
no	51	44%

2) Do you know what the vaccine is given for (asked to those who said they had heard about it)?

Yes	37	57%
No/incorrect	28	43%

3) If yes how did you hear about the new vaccine (some mentioned more than one source)

Church	Clinic	Don't know	Media	Neighbors	Newspaper	Radio	School	Tv
1	11	3	3	1	1	5	1	43

Day 2

Total asked: 83

1) Have you hear that there is a new vaccine for babies 0-11 months?

Yes	53	64%
no	30	36%

2) Do you know what the vaccine is given for (asked to those who said they had heard about it)?

Yes	26	49%
No/incorrect	27	51%

3) If yes how did you hear about the new vaccine (some mentioned more than one source)

At the launch	Church	Clinic	Don't know	Family	Media	Neighbors	Newspaper	Poster	Radio	School	Tv
1	0	5	5	2	5	4	0	1	4	1	24

Total Day 1 and 2

Total asked: 199

1) Have you hear that there is a new vaccine for babies 0-11 months?

Yes	118	59%
no	81	41%

2) Do you know what the vaccine is given for (asked to those who said they had heard about it)?

Yes	63	53%
No/incorrect	55	47%

3) If yes how did you hear about the new vaccine (some mentioned more than one source)

At the launch	Church	Clinic	Don't know	Family	Media	Neighbors	Newspaper	Poster	radio	school	TV
1	1	16	8	2	8	5	1	1	9	2	67
