# Supplementary material for: Stakeholder perceptions of communication about vaccination in two regions of Cameroon: A qualitative case study
Source: PLoS One. 2017 Aug 31;12(8):e0183721. doi: 10.1371/journal.pone.0183721 (PMC5578665; doi:10.1371/journal.pone.0183721)
Supplement: S4 File — (PDF) [file pone.0183721.s004.pdf]

Field notes

Vaccination Campaign in Yaoundé

Thursday 06.02

Followed a team for the first day of the campaign. Visited three schools and went door to door. One on the team was supposed to be a social mobilizer but I did not see that she did anything different from the vaccinators. I don't think that the schools were informed we were coming. We also vaccinated any children we met in the street even if their parents were not present. When entering a class room all we said was hello and good bye. "T" always said the vaccination was for polio but I am not sure the others did.

Met the team at the hospital in the morning. It seemed fairly unorganized. People were unsure of where they should be going. We got a few blocks away before we realized that we had forgotten to take the vaccines with us. Out first stop was a private school. We got there just in time as the students were all getting ready to leave for their sports lessons. The principal did not know we were coming and he did not realize that there would be 3 sessions. Many thought that since they had been vaccinated in January that they were finished. This was a common theme throughout the day. "T" handled it better by saying capaigne deuxieme visite and then explaining that there were three. I am not sure the other group made that distinction.

When we arrived at the school the children were just told that they were going to be vaccinated. They were not informed what against. I am unsure if parents knew what was happening at the school. This is definitely a missed opportunity to talk to kids even for a few minutes about what is going on. All the children were very obedient and only one spit out their vaccine. The teacher made them take it again. (I will ask tomorrow if there was anything that was done differently the first time around)

After the schools we went door to door again there was not very much conversation just that it was for Polio and that there would be another round in March. There seemed to be no real pattern to where we went just wherever we saw kids. If we walked by a kid on the street sometimes they were stopped and sometimes they weren't.

I did not see any social mobilization to speak of although we had a mobilizer with us.

Field notes from field journal:

- Meet at the hospital and collect vaccines
- "Q" says that there have been announcements on the TV and the radio
- Thursday and Friday we will focus on schools. Saturday and Sunday is more door to door to catch those we missed in the schools and those who do not yet go to school.
- I am told that the schools know we are coming
- There are 5 teams and each is given a different neighborhood each day
- We caught the first school just as they were going out. They were walking in straight lines in their gym uniforms
- The chef did not seem to know we where coming and some negotiations took place so that we could go ahead and start then.
- Apparently some children don't know how old they are. That is what one teacher said
- There is some confusion over what age will be vaccinated
- Viles are kept in a cooler and then pulled out. New dropper is attached to each vial

- "A" starts at the beginning of one line (150 kids approx.)
- This is a private school
- Once the child has been vaccinated their pinky finger is marked with permanent marker
- Once they have received the vaccine they go into a separate classroom where "K" marks them
- "K" and "A" are in white Min Sante pinnies and I am in a yellow one that says Unicef and Min sante on the front and social mobilizer on the back
- All of the kids, especially the older ones, are making faces and saying it tastes sour
- Some smaller neighbor children also come to the school once it is known that we are there
- There is no information given on what Polio is or why this is happening to them
- One the kids start getting rowdy the teacher says be proud to have visitors comportez vous bien et silencieux
- Children are counted as they leave the class room and the teachers are asked how many are absent today
- The group is extremely well behaved for 3-4 year olds
- If we had come 5 mins later we would have missed them
- 47 maternelle and 70 older
- Head tipped back, hold mouth, count drops
- The white pinnies on the front say Vacinons nos enfants with Min Sante and the logo
- On the back they say: La vaccination est gratuite pour les enfants do 0-11 mois, Min Sante
- "A" finishes with the school and does the neighbor children
- They then record the number of vaccines used on their sheet and finish marking the fingers
- The sheet is divided into 3 age groups (0-11 months) (12-59 months)(5-10 years)
- There is a man across the street on an old trundle sewing machine and above him the marks from the last campaign stand on the door
- One student goes home and brings back her younger sister to be vaccinated
- The second school is much bigger. A mother asked us to vaccinate her kids on the way down and they reply that they will do it on the way back.
- Start with the youngest class and work our way up
- Enter the classroom and go table to table. "A" drops and "K" marks. Part way through another vaccinator arrives name "T". I move to help her in a second class room where she does the drops and I mark fingers
- We take a break as the kids have a 30 min recess and a lot of the kids crowd around me to stare. I see a child with a spaghetti sandwhich. The others are playing a game that involves singing and jumping and looks a little rough.
- At the end of recess they form up into class lines and sing a song and then march into their classrooms. This is practice for the Journee d'enfance on the 11th
- 11h start door to door but not many children home. They are all at school so we decide to go to another school. On the way we vaccinate some children. Everyone accepts except for one father who repeatedly ignores three attemptst to greet him and make eye contact. One mother mentions hearing something yesterday about the campaign and that this is the 2nd of three days
- The third School
- At first the principal thinks we are only here to vaccinate the kids that were missed last time. It takes some time to explain to her that we want to vaccinate all of the children and that they need three doses to complete the vaccine. The Maternelle classes are away so we will return for them tomorrow but we do all the others.

- We need to come up with a better way to count the number of children vaccinated. The counting just turns into organized chaos. When we leave the school we write on the door how many children were absent and how many were vaccinated with the marking T2 and an arrow in the direction of where we are headed next.
- We meet two girls in the street who refuse the vaccine. They are about 10 and say that their teacher said they didn't have to take it. They run away. A few houses down we meet their moms who do not agree and say they are going to talk to the teacher and spank the girls.
- Another little girl runs after us to get vaccinated
- After this we continue door to door for the rest of the day. This is "T"s' neighbourhood and everyone seems to know her. 99% of people are very welcoming once we say why we are there and even yell at their neighbours to bring out their children.
- At 14:00 we head back to the hospital to hand in our reports. I did not write down the tally but it was over 550 kids vaccinated from our group. We did not count how many houses we went to and how many houses where there was no one home. We guessed for the sheet. This is a very difficult exercise as the streets are not defined and at times it is hard to tell what is a house.

Friday 07.02

Today was similar but different from yesterday. Still seemed rather unorganized and I heard the same complaints from some of the vaccinators around the organization of the teams and who was supposed to cover which area. There was a lot of confusion about who was supposed to go with who. I spent the day with "K" and "F". We walked about 15 mins down the road from the clinic and then started going door to door. We did this until 1230. On our way down Mme "T" saw us and stopped to say hello and to reinforce the importance of marking the little finger on the right hand. She had some evidence that the wrong finger had been marked at a number of schools. "K" said that the issue had been raised in the morning meeting and we had talked about it as a team before starting out. We had a bit of a up down start as we wanted to go to the end of the road and work our way up and parents were stopping us along the way to get their vaccinations. The neighborhood was quite a maze and it was really hard to tell where individual houses were and it was quite easy to get lost and come up on the same house from the back from the next street over. The team today was much better at communicating with the parents. They always said, " Bonjour, campaigne de vaccination (sometimes contra le poliomelite) deuxieme dose. C'est pour les enfants de 0a 10 ans. Il y aura un troisieme passage en mars."

Field notes:

- Another slightly disorganized start. I arrived at 8:05 but "A" had already left. I went and waited outside for her to come back but "K" came down so I went in and joined the meeting and division of neighbourhoods. I left with him and "F" instead of waiting for "A".
- Many of the parents tell their children that the vaccine is a candy "bon bon" so that they will open their mouths. The vaccinators refer to it as this as well.
- 9 yr old girl and mother who almost refuses saying it is not necessary. "K" explains that the disease is not treatable and causes paralysis. It is better for the child's health to take the vaccine because if they get sick there is nothing that can be done. She accepts and the girl receives the vaccine.
- Some of the children we meet have washed off the mark from yesterday.
- Often neighbours inform us where the next children are especially in the windy house on top of house areas. If it were not for this we would never have found some of the children.

Especially the babies that are very small. Once the children are walking they are usually outside playing when we come by.

- At one house the uncle phones the mother to check and make sure it is ok to vaccinate. The phone is passed to “K” who gives the normal spiel and she agrees. The baby receives the vaccine
- As we are walking I ask about the training they received to be campaign vaccinators. They say that they had one day which mostly focused on the technical around cold chain and how to give the vaccine. They received a short training about what to say to parents who do not want to vaccinate. They used the words, “ il faut les amenes a comprendre”
- When we come across children today and we can not find the parents the vaccinators ask “on te vaccine souvent?”
- Today the door to door was much more difficult and maze like. Finished the neighbourhood at 12:30 but were told to go back out and double check the door markings that they earlier teams had done. We walked through the market and ended at “F”s house. “K” left and I stayed with F while she completed the paper work. We did 110 vaccinations at 99 houses with no refusals today although there were a few parents who needed more convincing than others
- I asked her about the communication training and she said the same as “K”
    - Try and explain that polio is not treatable, it causes paralysis and vaccinating is the best for the child
- when they went around in January they said that there were going to be 3 passages
- she said that there were also information on TV and Radion for promoting

**Saturday 08.02**

- Forgot to write in yesterday about the information shee that the soc mob have to show to parents who request to see information from the MoH (see Photo)
- Today we were double checking and marking houses and store fronts on the other side of the street from where we were yesterday
- I asked a mother if she knew that her child was going to be vaccinated at school. She said no that she did not know that it was going to happen this week but there had been Sensibilisation in January and some thing about it on the TV. She seemed fine with not knowing when it was going to happen. She thought that the son had been vaccinated at school but when he came to the door he had not so we vaccinated him.
- It is starting to be hard to tell which kids have been vaccinated as they are washing off the marks. The same with the houses. It would be good to explain the reason for the marking of fingers and houses at the schools and when teams go door to door.
- A child does a happy dance when we mark his finger
- Children seem really proud/ Happy about their finger markings
- Kids show us their fingers in the market and say “on m’a vaccine”
- Multiple levels of communication
    - Fingers
    - House marking
    - Talking
    - Pinnies
    - All with different targets and purposes

- “K” said that last time they gave an announcement at the church and the team waited outside on Sunday
- One grandmother asked us for her vitamins as the last campaign was vitamin A
- 80 vaccinations today

Parents who refuse at first or seem apprehensive get more explanation. This was made clear when we met a poor mother with 5 children who was very apprehensive about vaccinating the kids. She said that they all got sick last time (I had the feeling that this wasn’t true by the way she said it but am unsure) “K” took the time with her to explain what polio was and why it was important to vaccinate, that there had been no reports of kids getting sick after the vaccine in January. After some discussion she agreed. Most parents accept right away even before you say polio. The word vaccination is enough.

I had the feeling today that the team was tired. We only walked half way up the hills and did not get near the top of the neighbourhood all though we could see children at the top. I did not want to influence their decision so kept out of it.

At the end of the day we went up through the old market and double checked an area that “J”was pretty sure had not been covered. After that we sat down in a small store and I bought them a cold drink. We chatted about their lives and what they were doing. All had taken the one day training.

Again today they were holding off before heading back because they didn’t want to be sent out again. I am thankful that I was not working in Douala. It was hot enough here. In the morning while they were deciding where teams would go and waiting for everyone to arrive there was discussion that the benefits and pay were better before but that now it is worse. By the time the finds trickle down to the people going door to door they have the feeling that each level above them has taken their cut.

**Sunday 09.02**

Arrived at 8 am and was the first one there. It felt like Sunday and everyone’s energy was starting to lag a little bit. There was a lot of talk about god and church and being a good Christian. I was sent out with “K” and “J” again to double check an area called la passerelle and then go to the Catholic Church. On our way we saw an OMS vehicle and “K” said they were probably controlling the area i.e. making sure all doors were marked. Today we relied a lot more on the public to tell us where had been covered and not covered. A lady pointed us down a short road that had not been covered and we ended up vaccinating 8 kids there. One of the children we vaccinated was done before we could talk to the mother as she was in the shower. She came out and called us back afterwards slightly concerned and a bit frustrated as the son we had vaccinated was 11. He had not told us his age but had just said he had not been vaccinated at school. His sister had. The mother was concerned that because he was 11 that the vaccine would be harmful. Once “K” reassured her that this was not the case she was fine. We continued up the road and met lots of people heading for the church. Two mothers stopped us on the way. Once we arrived at the church we sat down in the shade and let people come to us while controlling the children directly around us. After a while “K” and I walked around and check out the 200 kids in the church yard. Then we sat down and waited again. The Pasteur (older white gentlemen) came over and told us we should go stand by the door to make sure we got everyone as they came out. When the service let out this is what we did. He said that they had been announcing the campaign in the church in the week leading up to it. When people were coming out of the church it was a bit chaotic. And hard to track and mark all the kids. There were a few of the older children 8-10 years who took it upon themselves to become finger checkers and

bring or point out to us any one who had not been vaccinated. One girl even brought as a woman and her baby who had just been walking down the street. Once the next service started things quieted down a bit. A few more kids came over but then a nun started chasing everyone away. At 12 we headed back to the hospital and dropped off the left over vaccines and our form with the number of vials used on which age groups. (80 vaccinated)

- Target the churches and religious ceremonies
- Eglise catholique, annonce pendant la messe
- Discussion about which zones still need to be covered. With no road names this is done by landmarks and neighbourhood names
- Need to know the neighbourhood well or else you would not know where you were going
- There was a long discussion/argument about the door marking as rachelle was saying that the teams weren't doing it while other team members were saying people were washing it off with soap and water and that is why there was no marks.
- There is talk about teams being sent out again on Monday night to do a catch up
- In front of the church hald heartedly and randomly checking kids that come close enough. Calling them by mes amies
- Went into a quartier for 30 mins and found about 10 kids
- I think that we are waiting for the church to let out
- Vaccinating while waiting
- Pasteur says that they announced it in church
- Children go out and find a mother and bring her back to us
- Word of mouth that we are in front of the church so more people come
- Now waiting again as the next service has started
- The biggest communication strategy seems to be the bibbs.

# Campaign observations Fundong health district

## Notes from Mabel

We left at 7:15 to the district hospital picked up ma “S” and on our way to Belo.

Arrived Alim ma “S” had forgotten vaccines and so we went back to the DH waited for about 20 mins for the DMO to show up and let us in and got the vaccines. Finally left for Belo.

Arrived Sho received a call from district officer that vaccines had to be delivered to Kikfuini. So had to go back for delivery

8:45 delvered vaccines to Kikifuini and left for Belo Health centre

We left 20 vials with them for a total of 150 vials

From there we left to Mejang which is below Mbingo. We met the head of clinic who said that routine vaccination is small so is done monthly as outreach and they have no fridge.

Then we met the newborn baby.

On the way down the road we met with two vaccinators and gave them a ride to their next school. We met them after they had vaccinated 11 children at first school. We went to the second school 0-5 years old children assembled.

Vaccinators said mothers are informed when they deliver at the clinic and every time they visit the clinic they are told about vaccination. There are no drop outs because mothers area informed of the importance of the vaccines. Other forms of communication include clinic outreach and social mobilizers who move from door to door. Most students at the school had not come because it was a Friday and some did not come because they were adjourned for not paying their school fees.

The male vaccinator marked the children's fingers while the female vaccinator gave the vaccine. Once a child had been vaccinated and marked they went into the classroom. Once the children had been vaccinated the male vaccinator marked the window of the class room saying that 8 children were vaccinated and 11 were absent.

10:23 left school and head to Anyajua

10:51 arrived Anyajua and the doctor was in a consultation so we waited. We said hello to the second woman who was record keeping in the office who had rushed in from the farm to help. I asked "S" about the vaccinations for this clinic and she said it is once a week because they have a fridge. .Dr. said they started with the campaign yesterday. They started with a briefing. Today all of the vaccinators were out in the field. We saw the communique to churches and CBO about the campaign that the head of the health area had sent out on February 28$^{th}$ and had been read out at meetings, schools and church services.

"S" inspects the hospital, checks the fridges for organization of the vaccines, labelling and temperature recording. Then she signs the doctor's consultation record book. I asked the doctor if other health areas send out communiques like he does. He replied that he believes so and thinks Belo did. I ask if he received any feedback from the communique and he said that the vaccinators say that it was read in churches. His vaccinators had left already and were moving from school to school.

On our way down we met with a vaccinator from another clinic who had run short of vaccines and so was going to pick some up

11:35 left clinic and went to the second health clinic in Nayajua. The nurse in charge said that all vaccinators were out. S asked if there were any new cases of measles, YF or TT and the nurse replied no. We checked a child waiting outside and they had not been vaccinated. The nurse said that he is still saving for his fridge and has 300 000. He was asking how much the fridge cost and S said she did not know but told him what he needed to have in the fridge. He does vaccines once a month because he has no fridge. I asked him how mother receive information and he replied that they receive info from the district through meetings in the health areas (I think he was talking about how he receives information not the mothers). I asked about EPI and he said when they go for outreach programs and every time the mother comes to the clinic they tell them. The ward were neat as compared to the hospital before except for the maternity room which he said someone had just left and the cleaner had not yet come. He showed us the EPI hall and the charts for the vaccinations and ANC. I asked him if he had any problems with drop outs and he said no. I asked do most children finish their vaccines and he said yes. I asked why and he said because they know the importance of the vaccines. He then explained his charts to us. I asked if the completed vaccination rate was for the 9m vaccinations and he said yes. Have to indicated this because of performance Based Financing. There are 35 indicators that they have to fill in. I asked about the population base for the clinic. He replied 3500. I asked how many health workers and he said 4. One is a diplome nurse, a midwife, an auxiliary lab tech and a cleaner. As we were leaving he gave us a Pineapple.

In the car I asked S when EPI came to this region. She was unsure if it was in the 80s or 90s.

On the way down the hill we stopped at a private Christian health centre to check on their campaign activities. The vaccinator we met on the road came back and had received vaccines. The nurse that ran the clinic said that they had finished in the schools. The nurse said that they were waiting for the rest of the children to come and take the vaccines because the teachers said they were dry cleaning and they would come when they finished. S asked about any cases of VPDs and the nurse replied no. S signed the register. I asked if there was a fridge and there was no fridge. Took a picture of the old campaign poster from last year

12:00 left for Ndaware stopped for lunch in Belo¨

13:30 arrive Ndaware We met with a nurse who had not gone out on the campaign today. I asked about the EPI schedule but S did not know. The nurse was trying to call the doctor but could not get her. The clinic has two fridges. I asked about the population of the town but she was unsure as it fluctuates based on time of year so they had allocated 2000 doses. S says campaigns are a very tiring job that you feel like you need to rest for at least a week afterwards but the pay is poor. She does it just to help. The vaccination part is just the worst and that most nurses stay away from it (I think she means it is tiring and unpleasant as children squirm) While waiting until 14:30 when S and I vaccinated a toddler who had been waiting since we arrived. The little girl struggled. Then we went for a walk and got some tea. Came back to the hospital  and decided we could not wait any longer. She dropped the forms and I reminded her to check the fridge. They were running out of vaccines so we left 15 vials. She requested than any remainders be brought back to Fundong.

15:00 leave Ndaware for Njichami. Arrive 15:39 Met with the nurse. S marked the register and checked the fridge. Nurse said they heard about the campaign only yesterday. S asked if they had started and she said that they started today. S said that is no problem the most important is that they have started. The power had been out for three hours so she checked the temp.

Left at 15:44 for Belo Health Centre where we deposited forms and checked the vaccines to see how many were left

Collected plantains and tomatoes and continued on to Kikfuini. The nurse had not completed the tally sheets so S sad to send them to Fundong and we left.

On the way home we stopped at Funantui sub district hospital to pick up tally sheets but they were also not ready

## My notes

### 07.03.14 Campaign

Arrive 7:30 pick up S and leave and then Belo calls to request more vaccines so we turn back to the HC and wait for the DMO to come and open the door so we can take the vaccine. Once he arrives we load the vaccines for Belo. One was yellow so we left it behind.

8:07 leave. Tah says it is easier if we do the towns in the reverse order of what S suggested so that we start with the one that is farthest away and then work our way back.

8:37 call from doctor asking if we had already passed K health station. S says yes but he asks us to turn back and drop off more vaccines there as well. We dropped of 200 doses and then left another 100 doses for good measure this leaves a total of 14000 for here now. We were going to leave droppers but had brought the wrong size. They will just have to manage with what they have. We leave chalk as well. There is a green map on the wall titled Kikfuini itinerary maps for NID Polio which shows the walking routes for the vaccination team.

8:47 leaving S says there are a number of clinics that are short on vaccines because of the way they were distributed this time around. She says that that is one of the reasons that we are doing the rounds of some of the health centres today. There is still confusion over the number of Social mobilizers each clinic can use as they have not been able to open the budget from UNICEF. S says she is not sure what they would do without us and the car. We are making life easier.

All of the HWs we have met are wearing marked T shirts. They are not wearing bibs like in the city. All of the t shirts are not the same. They are from various past campaigns but they all say min sante and Unicef. S's is from the African vaccination week.

I have the sense that the change of date in addition to women's day has really confused things. Some clinics started yesterday to give the women Saturday off. This seems to have affected training and organization along with the delivery of the vaccines.  I must ask the DMO about the training manual.

9:02 dropped off 20 vials at Belo Health Centre for a total of 150. They said that they should be fine until Sunday now.

9:20 passed Mbingo Hospital

9:40 Arrive at Mejang outreach clinic. They do vaccinations once a month as they have no fridge. I ask the head of the clinic how many mothers they currently have doing their vaccinations at the clinic and she says 12. She asks S about a missing form for the cost of the transport of the vaccinators. S says they did not receive one and she is unsure what they should do. I asked the health person how they communicate with mothers in this health area and she said every time that a mother comes to the clinic they talk to her about vaccination. They have a very low drop out rate. I wonder if this is because it is such a small tribe and that they know everyone so they can follow up. There is a mother at the clinic who has just delivered. The head says it is the first delivery in three months. She also show us the new toilets that have been built but that are not yet being used as there is an issue with a crack in one of the tanks. The clinic is very neat and tidy. We thank her for her time and head back towards Mbingo.

On the road we meet a vaccination team that has just finished with one school and are walking to the next. We give them a lift for about 10 mins which saves them a lot of time. I use the opportunity to question them about communication activities in the area. The female vaccinator works at the health clinic we just visited. She said the main way in which they give information to mothers is through talking to them at the clinic. These are not necessarily organized health talks but each time a mother comes they talk to her about the importance of vaccinating. They do give health talks on vaccination days. She says there is a very low drop out rate. I ask her if there are any other ways that they communicate with mothers in the area and she said that they also use social mobilizers who go door to door and present at community meetings. I asked her if the fact that it was a small town made social mobilization easier as everyone would know everyone and if a new baby was born it would be news. She said yes. I then ask her how the campaign is going and if she has met any resistance. She says no the campaign is going well and that the only confusion is over the age since the last two rounds were for 0-10 and this one is 0-10. She also says that a lot of parents are saying again? She was unsure if this was going to be the last round or not.

We arrive at the school and start walking towards the nursery wing. She says to the children "lets go back to class". We arrive at the classroom and the teachers start dividing the children by age. The vaccinator says "just help us like last time" There is some confusion with the change of age for this campaign. I ask if I can take pictures and they say yes. The vaccinators line up the children who are to be vaccinated. There are 8. The male marks the fingers and then the female gives the vaccines. After

that they are sent into the classroom to wait. Once the vaccinations are complete the male rights on the wall of the school and asks if many children were absent. The teacher replies yes as it is Friday and also a number have not yet paid their school fees. I look in the classroom and all of the kids are comparing their fingers to see. They seem proud of their markings. We walk back to the car and say goodbye to the vaccination team and start back towards Belo.

10:30 we pass a vaccinator talking to a mom with two small children on the side of the road.

11:10 We arrive at the next clinic. It has become clear that S is also doing her monthly supervision visits. She checks the fridge for how it is labelled and the temperature. She asks if there have been any outbreaks of VPDs. She then signs the clinic book. At this hospital there is a letter that the chief of the health area has sent out to schools, churches and CBOs. I ask to take a photo and ask him some questions about the letter. (see notes above for conversation)

This chief also asks S about the number of Soc mob that he can use and again she has to say that the funds have not yet arrived. He says that he received 200 vials and 166 are already out. He also started yesterday because of women's day on Saturday.

From now on during the day Mabel is taking the notes and I am taking pictures of the various communication materials in the clinics and asking questions.

## 08.03.14 Women's day grandstand and parade

We arrive at the grand stand at 10:10 and take our seats. We buy yellow ribbons to support women in council. The district officer arrives and then the speeches start. The Mayor mentions the MDGs in his speech.

- An economically empowered woman has already achieved all of the MDGs
- The usefulness of health education to reduce child mortality and unsafe sex which would decrease HiV
- The bible says that the man is the head of the family and you as women should respect that as such so we can achieve the MDGs together
- Celebrate today responsibly

Now the divisional delegate for women's empowerment and the family has her speech

- She also talks about the MDGs and says they are the theme of women's day "Challenges and achievements of the MDGs for women and girls"
- The UN is asking for a pre evaluation of the MDGs and proposals on how to overcome the obstacles that are still being faced
- Talks about MDG 3 gender equality and education and that this goal cuts across all of the MDGs
- The public are being distracted by the police who are redirecting motorbikes and took one away from a driver
- The speech goes on to talk about the roundtable discussions that were held last week and the challenges still being faced in Cameroon. These include:
    - Ignorance from illiteracy despite the increase in school enrollment
    - Not all girls attend school
    - Young marriage
    - Girls come to the office to complain but cant file their complaint in English

  - Family violence and spousal abuse
  - Rape
  - Power of traditional institutions that do not allow women to inherit and the problem of wife inheritance
  - Poverty as a woman cannot own or inherit land so cannot get credit
  - Refunding of dowry by girls who want to get out of their marriages
  - 50 recorded pregnancies in the schools of which 10 were HiV positive
- While she is talking some of the important men are talking between themselves
- Girls should join the school health clubs to learn about good health and healthy relationships

Then there is a summary of her speech given in Kom

The next speech is the head of all of the women's organisations in Boyo

- She also mentions the MDGs as it is the theme for this year
- Calls on the women to dress responsibly as the way you dress reflects you

Summary in Kom

Next is a word to the women from the senior divisional officer of Boyo

- A day to reflect on the numerous challenges faced by women
- In the domain of health
  - Neonatal health and delivery
  - Breast and cervical cancer screening thanks the DMO
  - He is aware of the difficulties faced by the health services
  - Talks about blood donation
- Talking about how land is passed and the challenges of tradition
- Reminds the audience that law remains above tradition so denounce mal treatment
- Talks about the case of widows and says that we should safe guard estates for them and their children
- Call for education to build a profitable partnership and says women your place is no longer behind
- Honorable clap is given (3 pause 3 pause thunderclap)
- He calls on the men sitting in the grandstand to take the pledge "I will not be an obstacle to the development of the women"
- Another warning against drunkenness

Now there is an animation with singing and dancing (see video) the song is about the MDGs

Then the march past of all of the different women's groups in Fundong. Many of the groups carry posters with health related slogans

After the march past a number of the different groups perform. If people appreciate the performance they go up and give them money. The most popular was a traditional song and dance group lead by an old granny who said funny things into the mic and stuffed the money in her pocket instead of letting people put it in the bucket.

In between the acts an announcement about the vaccination campaign is made

- The health team is inviting all children 0-5 years to come for vaccinations who have not yet been vaccinated to the right side of the grandstand. The campaign against Polio will go until Monday.
- Repeated in Kom
- Let us vaccinate our children

Mabel and I go over to the side of the grand stand and meet the vaccinator. She is the one with the bun from the UHC. “A” is there as well. A number of mothers are coming over and in between she watches the show. As mothers pass we ask them if their children have been vaccinated and as children pass we check their fingers. We ask a few mothers how they heard about the vaccinations. Some say the loudspeaker announcement, others say word of mouth and that they also heard on the radio yesterday. Anna and Mabel go out and walk around to try and find people. One mother heard the announcement but did not come until Anna sent her.

One mother that Mabel talked to was reluctant as she had not heard about the campaign from any source but finally, reluctantly let her son take the vaccine. “A” asked if he goes to school and she replied no.

In total, while we were there 46 children were vaccinated.

18:10

Watching the women’s day march from Yaoundé and the Ministry of Public Health is marching by. They are talking about the MDGs and mention the ongoing polio campaign for children under 5 as well as various other programs for Malaria and essential medicines. The announcer says that they should just go back to old methods of malaria control like avoiding standing water and then mosquitoes won’t come near you and you don’t need to sleep under a net. A pregnant woman marching underneath a treated mosquito net.

## 09.03.14 Last day of the campaign

### Heather’s field notes

7:45 on the way to the UHC we pass a vaccination team that has left the centre already. One vaccinator is carrying the ice box and the other is carrying a loud horn that when he presses a button plays a message about the polio campaign. We rush to the clinic thinking we are late. Anna greats us and assigns us to go out with “J” who is the focal person for Soc Mob for the Fundong Health Area. He has a yellow vest like the ones in Yaounde.

“A” says that she has 11 teams that she is in charge of. 3 in Fundong and the rest in 9 other communities. We are given 5 vials and asked to cover the Hausa quarter and some churches. We head out. We come to a house where “J” said the mother was complaining that she had been missed. We also meet a man who asks us what the campaign is for and then points us in the direction of another house with children who had not received the vaccine. He asks if children have been vaccinated by saying, “they don’t drop nothing for your mouth”. As we are giving the vaccine to the child that was indicated to us another mother finds us as she heard we were around. The babies are making very funny faces when they get the vaccine. “J” tells parents whose children are in Sunday School that they can bring them to the clinic tomorrow to be vaccinated.

I ask “J” if parents ever ask about why the campaign is happening, He replies that for the first round they did but now no as everyone knows when they see the dropper. I ask him what he tells mothers when they ask and he says that he explains that polio was kicked out of Cameroon but then there were 4 new cases in the fall in the neighboring region so that is why they are doing the campaign.

I think that the campaigns may be a missed opportunity to question and remind mothers of 0-11 month olds about their routine vaccinations.

After this Mabel and I switch. She has been marking fingers and I have been note taking but it is better for her to take the notes as she can understand the conversations.

A number of children we meet said that they had been vaccinated at the grand stand yesterday. I recognize some of them. One of them starts to cry when he sees me.

## Mabel's field notes

Ways mothers said they had heard about the campaign

| Social mobilizers | Radio | Neighbor | TV | Church | Hospital | When we showed up |
|---|---|---|---|---|---|---|
| 4 | 1 | 2 | 1 | 1 | 0 | 2 |

On our way to the Hausa quarter a woman asked us which vaccine it was and "J"replied Polio 0-5 years

Once we arrived in the quarter the 2nd vaccinator appeared and carried the cooler.

The first Muslim woman we visited was a revisit as the vaccination team had been there yesterday but had run short of vaccines. We kept going door to door and most mothers were telling us that their children told them they were vaccinated at school. The vaccinator verified that the children had been vaccinated by checking their fingers. We went to the small Muslim school in the quarter but all of the children were older than 5.

We asked some mothers and they didn't know if their children were vaccinated or not until the vaccinator checked their fingers. We went to another compound and the children were not there so the mother went and searched for them. We met a baby who had missed the first round but was vaccinated today. The next compound we went to the mothers were confused over which dose it was and said their children had missed the first dose. We double vaccinated a child as the mother said he had not received a third dose but he had received on Thursday and had missed the first round.

The local social mobiliser reminds "J" to write on her door even though there are not children and takes us to a neighbors house with a new born baby.

We went across the bridge to a house that "J" knew the child had been missed, The mother was completely uninterested and the older brother brought us the child and held her. The mother did not respond to "J" when he asked her a question. We then walked back across the bridge towards town and stopped at the deeper life bible church where a church member sent children out to us and then brought the smaller ones out. He said that there had been no announcements in church concerning the vaccination.

After the three corners we met a mother from Abu who said she had taken the first two in Abu but the campaign had not yet started there so she took the vaccine here and the vaccinator asked her to not take the vaccine again when they come to her house in Abu.

We left into the teacher's quarter. "J" said that mothers hear that vaccinators are passing by even when they are in their farms they don't bother to bring out their children and when they have missed the vaccines they still don't bother to follow up but then start complaining later on.

“J” says to the lady who was taking us to her compound, we are doing the revisiting because if we don’t they will say we have not done our jobs and I and “A” will be held responsible.

Went to her compound but the children had all been vaccinated without her knowledge.

We took a short cut back to the UHC and dropped off the avocadoes that the woman had given us. Then we carried on towards town to check the churches. First, we went to the smaller Baptist church and found 1 child that had not been vaccinated. Then we visited a house beside the church and found another child. Then went to the bigger Baptist church and there were many children outside. They were still in service but all the children we checked outside had been vaccinated.

Next, we went to the Catholic Church. The service had ended and we found several cases of children who had not been vaccinated. We checked the fingers to see and if not then we vaccinated. Other children assembled as they saw us and brought smaller children to us. Mothers called their children for vaccination. The second vaccinator said that we should hurry up as she has things to do today and she is only doing this because of the chief of centre who will say you like to spend money but you don’t like to work for it, If they have to return to the hospital early.

From the Catholic church we headed toward the market and checked children on the way. We found some cases in the market and vaccinated them and checked others to confirm that they had been vaccinated. The vaccinator had to call for mothers to verify if the babies had taken the vaccine. In the market a mother and a grandmother asked why the vaccines were only for children.

From there we went to the quarter head’s compound and vaccinated the children who had not been vaccinated and rested while they went out to bring in others they knew of. The mother said some of the children escaped yesterday and that is why they were not vaccinated. The mother asked why the vaccines are only for children. A mother sees us going into the compound and brings her child in to be vaccinated. The child we were waiting for finally arrives, says he couldn’t take the vaccine because he was driven from school because he had not paid school fees. From there we returned to the health centre. “J” agrees to meet us for an interview tomorrow.

# Media transcripts and description

## EPI office running add for new Rota Virus Vaccine

21.03.14

Under the great patronage of the great first lady of Cameroon, mme Chantal Biya, the ministry of public health introduces a vaccine against diahrea caused by rotavirus. Through the national vaccination program this vaccine will be given orally to children aged six weeks to 11 months. The vaccination against diahrea caused by rotavirus wil debut the 28th of march 2014 at all health clinics public and private and it is free. Together we will fight against diahrea caused by rotavirus.

## SMS received about new vaccine

29.03.2014

A PARTIR DU 28 MARS 2014, FAISONS VACCINER GRATUITEMENT NOS ENFANTS DE 0 A 11 MOIS CONTRE LES DIARRHEES A ROTAVIRUS. MESSAGE DU MINSANTE.

# Website reports about polio:

CRTV

## UN CAS DE POLIO SIGNALÉ À KRIBI

**12/03/2014**

**MUH MINATOU, FILLETTE DE 4 ANS SOUFFRE DÉJÀ D'UNE LÉGÈRE PARALYSIE DES MEMBRES INFÉRIEURS**

C'est lors d'un récent séjour à l'hôpital baptiste de Bansoh dans le Nord-Ouest que la maladie a été dépistée chez la jeune fille. Les parents de Muh Minatou l'y avaient conduit suite à une déformation de la jambe qui résistait aux massages. Elle est scolarisée dans la cité balnéaire depuis le début de cette année scolaire 2013-2014. La poliomyélite est de retour au Cameroun depuis l'année dernière. Elle avait été dépistée dans le district de santé de Bangourain. Certains font d'ailleurs un lien avec le cas découvert à Kribi puisque les parents de Muh Minatou sont originaires du département du Noun.

La nouvelle de la découverte de ce nouveau cas mobilise depuis quelques jours les autorités sanitaires. Alim Hayatou, le secrétaire d'Etat à la santé publique s'est rendu à Kribi en compagnie des responsables des services compétents du ministère de la santé. Les responsables de l'OMS étaient du voyage. Ils ont procédé à une large opération de vaccination des enfants de 0 à 5 ans de la ville. On a vécu une stigmatisation de l'école primaire où est inscrite la jeune Muh Minatou. La directrice de l'école avait refusé d'accueillir la dernière fois les équipes d'une campagne de vaccination.

**Elvis Mbimba**

http://www.crtv.cm/cont/nouvelles/nouvelles_sola_fr.php?idField=13214&table=nouvelles&sub=societe
